# Development and validation of the behavioral intention scale for participation in traditional Chinese medicine exercises for pulmonary rehabilitation among COPD patients

**DOI:** 10.1097/MD.0000000000045336

**Published:** 2025-10-31

**Authors:** Yuyin Chen, Meijiang Chen, Xiuhong Long, Huiqiong Tu, Yuanyuan Zhang, Jie Jin, Yuhua Qiu, Wanlin Peng, Shujin Cheng

**Affiliations:** a School of Nursing, Guangxi University of Chinese Medicine, Nanning, China; b Department of Respiratory Disease, Ruikang Hospital Affiliated to Guangxi University of Chinese Medicine, Nanning, China; c Department of Nursing, Ruikang Hospital Affiliated to Guangxi University of Chinese Medicine, Nanning, China; d Department of Gastrointestinal Surgery, Chengdu Seventh People’s Hospital, Sichuan Province, China.

**Keywords:** chronic obstructive pulmonary disease, intention, pulmonary rehabilitation, scale, theory of planned behavior, traditional Chinese medicine exercise

## Abstract

This study aimed to develop and validate the behavioral intention scale for pulmonary rehabilitation with traditional Chinese medicine in COPD patients (BISPTCM-PR-COPD), based on the extended theory of planned behavior. Following the COSMIN framework, the scale was developed through literature review, semi-structured interviews, and expert consultations. A preliminary item pool was refined using item analysis and content validity evaluation. Psychometric testing was conducted in 2 patient samples. Exploratory factor analysis (EFA) and confirmatory factor analysis (CFA) were performed to determine the factor structure. Reliability was assessed via internal consistency, split-half reliability, and test–retest reliability (ICC). Convergent validity was examined using composite reliability (CR) and average variance extracted (AVE). The final scale contained 26 items across 5 dimensions: basic knowledge, attitudes, subjective norms, perceived behavioral control, and behavioral intention. EFA supported a 5-factor solution, explaining 68.9% of the variance. CFA demonstrated acceptable model fit (χ^2^/df = 2.259, RMSEA = 0.075, IFI = 0.941, TLI = 0.933, CFI = 0.941, GFI = 0.822). Reliability was strong, with Cronbach α = 0.951, split-half reliability = 0.883, and test–retest ICC = 0.927 (95% CI: 0.86–0.97). Content validity was satisfactory (S-CVI = 0.933). Convergent validity was supported, with CR values between 0.735 and 0.973 and AVE values between 0.362 and 0.901. Notably, the basic knowledge domain showed lower AVE (0.362), suggesting item heterogeneity. The BISPTCM-PR-COPD is a reliable and valid instrument for assessing COPD patients’ intentions to participate in TCM-based pulmonary rehabilitation. It can serve as both a research tool and a clinical screening instrument to identify low-scoring domains, thereby guiding targeted education and support strategies and facilitating more effective implementation of TCM pulmonary rehabilitation.

## 1. Introduction

Chronic obstructive pulmonary disease (COPD) is a preventable and treatable condition characterized by chronic respiratory symptoms due to abnormalities in the airways and/or alveoli, leading to progressively worsening airflow limitation.^[1]^ COPD patients are also affected by comorbidities such as cardiovascular diseases, cancer, osteoporosis, and diabetes, which lead to a decline in quality of life and an increase in mortality.^[2,3]^ Globally, COPD affects over 212 million people and causes more than 3.3 million deaths annually.^[4]^ Global projections indicate that COPD-related deaths may exceed 5 million annually by 2060.^[5]^ In China, nearly 10 million people are affected, with deaths expected to surpass 1 million by 2030, posing a significant public health challenge.^[6]^

Currently, COPD treatment primarily focuses on pharmacotherapy aimed at alleviating symptoms and reducing the frequency of acute exacerbations.^[7]^ However, the management of COPD also encompasses stable phase prevention, treatment, and home rehabilitation after discharge, highlighting the importance of proactive management.^[8,9]^ Nonpharmacological approaches, particularly pulmonary rehabilitation, have emerged as effective in managing COPD.^[10]^ This comprehensive, personalized intervention has been proven to alleviate symptoms, improve physical fitness, and enhance quality of life, establishing itself as a cost-effective COPD treatment strategy.^[11]^ It includes exercise training, respiratory muscle strengthening, nutritional support, health education, and psychological support, underscoring its significance in COPD management as endorsed by the American Thoracic Society (ATS) and the European Respiratory Society (ERS).^[12,13]^

Traditional Chinese medicine (TCM) pulmonary rehabilitation integrates the core principles of preventive treatment of disease, the unity of man and nature, individualized care, and a holistic view of health with modern rehabilitation concepts. Common practices include Tai Chi, Baduanjin, Wuqinxi, Yijinjing, Liuzijue, and pulmonary Daoyin, which are characterized by syndrome differentiation, root-cause therapy, and high patient acceptance.^[14–19]^ The Expert consensus and operational guidelines on exercise rehabilitation of COPD with integrating TCM and Western medicine emphasize that these exercises can alleviate respiratory symptoms, improve lung function, enhance quality of life, and reduce medical costs due to their low dependence on equipment, while remaining sustainable and practical.^[18]^ In COPD management, TCM exercises emphasize syndrome differentiation and root-cause treatment, aligning with the principles of preventing disease onset, halting progression, and reducing relapse.^[20,21]^ Overall, they are cost-effective, sustainable, and clinically effective, making them highly suitable for long-term application and broader clinical adoption.

Recent studies have shown that TCM exercises for rehabilitation face both high demand,^[22–24]^ and poor compliance.^[25–27]^ These issues not only limit their effectiveness in COPD management but also hinder the broader implementation of TCM-based pulmonary rehabilitation. Since individual participation largely depends on the intention to engage, understanding and assessing behavioral intentions is an important prerequisite for identifying barriers and designing tailored interventions.^[28]^

Despite the recognized benefits of TCM exercises, their clinical application remains limited by low patient engagement. To design effective behavioral interventions, there is a need for a tool that can identify patients with low intention and clarify the underlying factors, such as negative attitudes, perceived social pressure, or insufficient self-efficacy.^[29]^ Self-efficacy, defined as an individual’s belief in their ability to organize and perform the actions needed to achieve specific goals,^[30]^ is widely acknowledged as a key determinant of health-related behavior. However, to our knowledge, no validated instrument currently exists to measure COPD patients’ intentions toward TCM pulmonary rehabilitation, leaving an important gap between the demonstrated potential of these exercises and their practical implementation in clinical settings.

The theory of planned behavior (TPB) provides a robust framework to address this gap. By examining attitudes, subjective norms, and perceived behavioral control, TPB explains how psychosocial factors shape behavioral intention.^[29]^ It has been widely applied to predict diverse health-related behaviors, including blood donation,^[31]^ vaccination,^[32]^ health screening,^[33]^ and cancer screening.^[34]^ Given its explanatory power, TPB offers a solid theoretical basis for developing a scale to assess COPD patients’ intentions toward TCM pulmonary rehabilitation.^[35]^ Nevertheless, in the cultural context of TCM exercises, knowledge itself may act as a unique antecedent of intention. Unlike in other health behaviors where knowledge is embedded within attitudes or perceived control, here an accurate understanding of specific practices (e.g., breathing coordination in Liuzijue) is a prerequisite for forming meaningful attitudes and confidence. Therefore, this study extended the TPB framework by adding a “Basic Knowledge” dimension, resulting in 5 dimensions: basic knowledge, attitudes, subjective norms, perceived behavioral control, and behavioral intention.

The purpose of this study is to develop the behavioral intention scale for participation in TCM exercises for pulmonary rehabilitation among COPD patients (BISPTCM-PR-COPD; Supplementary File 1, Supplemental Digital Content, https://links.lww.com/MD/Q507), based on the extended TPB. This scale aims to visualize and quantify patients’ intentions to participate, providing evidence to raise awareness of TCM exercise use in COPD management and to assist healthcare professionals in identifying patient needs. Such information may support the optimization of medical resources and periodic service adjustments.

## 2. Methods

### 2.1. Study design overview

**Figure 1.**
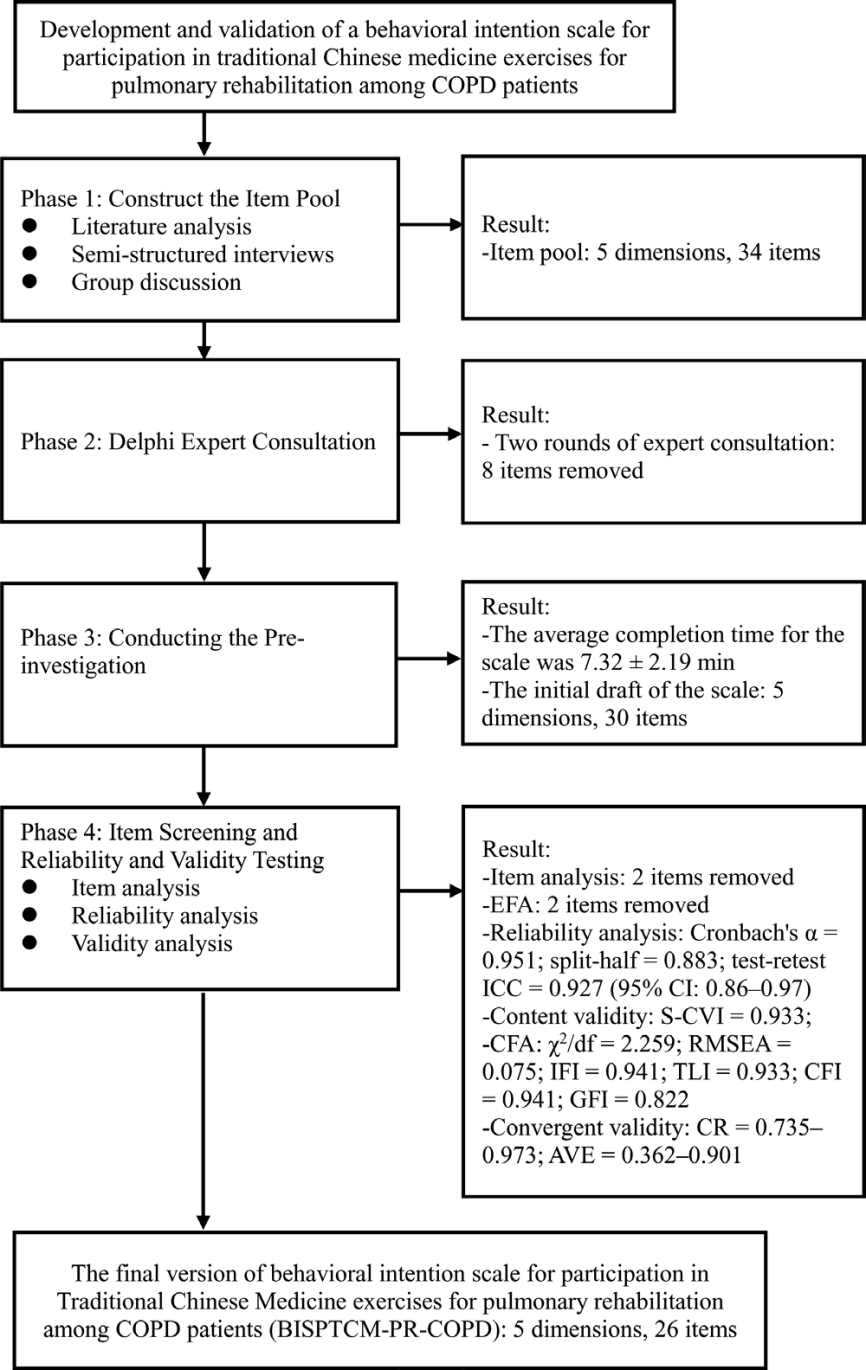
Flow chart of BISPTCM-PR-COPD development. BISPTCM-PR-COPD = behavioral intention scale for pulmonary rehabilitation with traditional Chinese medicine in COPD patients, COPD = chronic obstructive pulmonary disease.

To construct an instrument assessing the behavioral intention to participate in traditional Chinese exercises for PR among patients with COPD, this study was conducted in 4 phases (Fig. 1).

1. Phase I: Generation of an item pool through an extensive review of the literature, semi-structured interviews, and group discussions.
2. Phase II: Refinement of the item pool through 2 rounds of Delphi expert consultations to establish the preliminary items for the scale.
3. Phase III: Conducting the pre-investigation to refine the wording of the items and form the initial draft of the scale.
4. Phase IV: Formation of the final version of the scale through a formal investigation and evaluation of the initial draft of the scale.

### 2.2. Phase I: construct the item pool

#### 2.2.1. Literature analysis

A search for literature was carried out using 8 databases from 4 international (Web of Science, Cochrane Library, Scopus, PubMed) and 4 Chinese (SinoMed, CNKI, Wanfang, and VIP) databases to explore all literature related to this study. The search terms consisted of “traditional Chinese medicine,” “traditional Chinese exercises,” “intention,” “theory of planned behavior,” “scale,” “questionnaire,” “COPD,” “chronic obstructive pulmonary disease,” “evaluation,” and “assessment.”

The search of 8 databases generated 317 potentially relevant articles. It was comprised of 92 studies on the survey of people’s intention of TCM, 67 studies on the survey of people’s behavioral intention of the traditional Chinese exercise, 56 studies on the investigation to assess behavioral intention for patients with COPD, and 102 studies on the development of scales or questionnaires based on the TPB. An initial item pool for the scale was established, comprising 5 dimensions and 32 items, laying the foundation for subsequent semi-structured interviews.

#### 2.2.2. Semi-structured interviews

Based on literature analysis and the TPB, semi-structured interviews were conducted to gain a deeper understanding of the nuances associated with traditional Chinese exercises for pulmonary rehabilitation. These interviews aimed to compile and enrich the item pool further. For this purpose, 15 COPD patients were recruited. Informed consent was obtained from all participants, ensuring they were capable of communication and comprehension.

The interview outline of COPD was as follows: How familiar are you with TCM exercises for pulmonary rehabilitation? What is your perspective on engaging in TCM exercises for pulmonary rehabilitation? In your opinion, which individuals or groups might impact your decision to engage in TCM exercises for pulmonary rehabilitation? What factors or circumstances might influence your willingness to participate in TCM exercises for pulmonary rehabilitation? Have the decisions of others to engage in TCM exercises for pulmonary rehabilitation influenced your own consideration to participate? If so, who has been influential in this decision?

Based on the outcomes of the semi-structured interviews, researchers supplemented the item pool for the scale, adding 4 items, thus expanding it from the original 32 items to the current total of 36 items. All interviews were audio-recorded, transcribed verbatim, and subsequently analyzed using thematic analysis. Key themes and representative patient expressions related to the TPB constructs were extracted to ensure the final item pool had strong content validity from the patient’s perspective.

#### 2.2.3. Group discussion

Discussions among all group members after the literature analysis and the semi-structured interviews were conducted. Following discussions during the project team meeting, 2 items were revised, and 2 items were removed. The meeting also established the use of a 5-point Likert scale for rating. This resulted in a final item pool comprising 5 dimensions and 34 items.

### 2.3. Phase II: Delphi expert consultation

After the item pool of the scale was generated, at least 15 experts were invited to review the items and dimensions to score their importance and relevance and put forward comments for modification. The research group sent an expert consultation form to experts, which included information about the experts, judgment basis (Ca), familiarity with the theme (Cs), and the importance of each item. The importance score ranges from 1 to 5, with higher scores indicating a greater match between the item and the scale.

Criteria for Expert Selection: a minimum of 10 years of professional experience in the fields of Traditional Chinese exercises, COPD, pulmonary rehabilitation, scale development, clinical medicine, or nursing; holding a senior professional position; attainment of a Bachelor’s degree or higher; willingness to contribute to this study by providing feedback.

We selected 15 experts from 6 provinces including Guangxi, Guangdong, Jiangsu, Anhui, Shandong, and Sichuan. The consultation form was sent to experts by email or WeChat and they were asked to give feedback within a week. Two rounds of expert consultations were conducted. After the first rounds of consultation, relevant data were extracted and analyzed according to the content of the feedback. We eliminated, modified, and added items based on the results of the data analysis and expert opinions to determine the second round of consultation. To avoid the effect of temporary memory, the intervals between the 2 rounds of expert letter inquiries lasted for more than 4 weeks each. After 2 rounds of consultation, the opinions of experts were basically consistent.

Four indicators were used to test the reliability of the expert consultation: the mean of the item importance and the coefficient of variation (CV) of the item; Kendall W; the participation coefficient for experts; the coefficient of expert authority (Cr). The item is removed if the mean importance score is <3.50 or the CV is greater than or equal to 0.25.^[36]^

### 2.4. Phase III: conducting the pre-investigation

To assess the comprehensibility and readability of the scale’s content, the preliminary items, refined through 2 rounds of Delphi expert consultation, were further adjusted and supplemented with instructions to create the version utilized in the pre-investigation. In June 2023, a convenience sample of 20 COPD patients was selected from the Department of Pulmonology at Ruikang Hospital, affiliated with Guangxi University of Chinese Medicine, to evaluate the understandability of the items and to ascertain the duration required for completion.

### 2.5. Phase IV: item screening and reliability and validity testing

#### 2.5.1. Participants and setting

A convenience sampling strategy was used to recruit patients with COPD from 4 institutions in Guangxi Province (Ruikang Hospital Affiliated to Guangxi University of Chinese Medicine, The First Affiliated Hospital of Guangxi University of Chinese Medicine, Guangxi International Zhuang Medical Hospital, and Liuzhou TCM Hospital) between July 2023 and October 2023.

Patient inclusion criteria: participants have confirmed diagnosis of COPD; age 18 to 80 years of age; participants are conscious and able to answer questions; participants are willingly taking part in the research and have signed the written informed consent form. Patient exclusion criteria: participants presenting with severe complications; participants with dementia or severe psychiatric disorders; participants with altered consciousness.

The sample size was determined using the formula: sample size = total number of items on the scale × (5 – 10). At this stage, the scale consists of 30 items. Accounting for a 10% rate of sample attrition, a minimum of 167 subjects is required for this investigation. For confirmatory factor analysis (CFA), the sample size should be ≥ 200 subjects. Accordingly, we plan to include at least 200 participants each for both exploratory factor analysis (EFA) and CFA phases.^[37]^

#### 2.5.2. Data collection procedure

Initially, the interviewer introduced themselves and inquired about the patient’s current well-being. They then detailed the study’s aims, the procedure for completing the questionnaire, privacy protections, and the estimated time required. Upon agreeing to participate and signing the informed consent, a personal interview was conducted. For patients with limited literacy or other barriers to self-completion, the interviewer read the questions aloud, recorded the patient’s responses, and ensured accuracy before marking the answers on the questionnaire. To avoid information bias, standardized language was consistently used. After submission, questionnaires were checked for completeness, and patients were requested to complete any missing or incomplete items.

Based on the data collected from the first cross-sectional survey, item analysis, reliability analysis, and EFA were conducted. Additionally, contact information was recorded for 30 participants during the survey, and they were asked to complete the same questionnaire again after a 10-day interval to assess the test–retest reliability of the scale. It was confirmed that these 30 participants did not experience any significant changes in their health status or treatment regimens during this 10-day period. Subsequently, a second cross-sectional survey was conducted, and the collected data were used for CFA of the scale.

#### 2.5.3. Instrument

1. General Information Questionnaire: This questionnaire, devised by the researchers after a comprehensive literature review, encompasses 2 segments. The initial segment collects demographic data, covering aspects such as gender, age, employment status, residence, cohabitation, marital status, educational background, monthly household income, and medical insurance coverage. The subsequent part gathers disease-specific information, detailing the COPD duration, presence of other chronic conditions, and hospitalization frequency.
2. Behavioral intention scale for participation in TCM exercises for pulmonary rehabilitation among COPD patients (BISPTCM-PR-COPD): The initial version of the scale consisted of 30 items across 5 dimensions. A 5-point Likert scale was used to rate the answers. All items are scored positively, indicating that higher scores reflect a greater willingness among COPD patients to participate in TCM exercises for pulmonary rehabilitation.

### 2.6. Statistical analysis

All data were coded and entered into SPSS version 29.0 (IBM Corp., Armonk) and AMOS version 21.0 (IBM Corp., Armonk) for statistical analysis. The demographic data of the sample and experts were analyzed using numbers, means, and percentages. In line with the COSMIN framework, this study evaluated several measurement properties of the BISPTCM-PR-COPD, including preliminary item analysis, content validity (expert review), structural validity (EFA and CFA), internal consistency (Cronbach α), split-half reliability, test–retest reliability (intraclass correlation coefficient [ICC]), and convergent validity (composite reliability [CR] and average variance extracted [AVE]).

#### 2.6.1. Item analysis

Four methods – CV method, Critical Ratio method, Item-Total Correlation Coefficient method, and Internal Consistency Coefficient method – were utilized to assess the discrimination and differentiation of the scale items, with items failing to meet standards in 2 or more tests being removed.

1. CV method: Calculated using the formula CV = standard deviation (SD)/mean, items with a CV <0.15 were removed.
2. Critical Ratio method: Independent sample t-tests were conducted between the top 27% high-scoring and the bottom 27% low-scoring segments, with items having a *P* >0.05 being removed^[38]^.
3. Item-Total Correlation Coefficient method: The correlation between each item score and the total score was calculated, removing items with a correlation coefficient (*r*) <0.3 or *P* >0.05.
4. Internal Consistency Coefficient method: If the overall Cronbach alpha coefficient of the scale significantly increases after the removal of an item, it indicates that the item is not homogenous with the other items in the scale, suggesting its removal.

#### 2.6.2. Reliability analysis

Reliability is an important indicator of the reliability and stability of a scale. In this study, 3 indicators, Cronbach alpha coefficient, split-half reliability and retest reliability, were used to test the reliability of the scale.^[39]^

1. Internal consistency: Cronbach alpha coefficient was used to evaluate the internal consistency and agreement between the scale and its items within each dimension. In general, a value >0.7 is considered to indicate good consistency.
2. Split-half reliability: Split-half reliability method was used to divide the scale items into odd-even parts according to the number, and then, the correlation coefficient of the score between the 2 groups was calculated. For a reasonable level of reliability, the split-half coefficient should be >0.7.
3. Test–retest reliability: Thirty patients completed the scale twice at a 10-day interval. Test–retest reliability was assessed using the ICC, two-way mixed effects, absolute agreement with 95% confidence intervals. An ICC value above 0.70 was considered indicative of acceptable stability.

#### 2.6.3. Content validity analysis

Content validity was assessed to ensure the scale’s items accurately represent the research theme. This involved evaluating each item’s relevance by consulted experts. The Item Content Validity Index (I-CVI) and the Scale Content Validity Index (S-CVI) were calculated. I-CVI is the ratio of experts scoring an item 4 or 5 to the total number of experts, while S-CVI equals the number of items rated 4 or 5 by all experts divided by the product of the total items and the number of experts. Acceptable content validity is indicated by I-CVI > 0.78 and S-CVI > 0.90.^[40]^

#### 2.6.4. Construct validity analysis

EFA was conducted to examine the dimensional structure of the scale. Data suitability was assessed using the Kaiser–Meyer–Olkin (KMO) measure (>0.70) and Bartlett test of sphericity (*P* < .05). Principal axis factoring with oblique rotation (direct oblimin) was applied, as the dimensions were theoretically expected to correlate. Factors were retained based on eigenvalues > 1, cumulative variance explained > 50%, item loadings ≥ 0.40 without substantial cross-loadings, and scree plot inspection. The factor correlation matrix was also examined to verify inter-factor relationships.^[41]^

CFA is employed to verify the congruence between theoretical models and empirical data and is a commonly used method for assessing structural validity of scales. A model is considered acceptable when the fit indices meet the following criteria: χ^2^/df < 3.0; root mean square error of approximation (RMSEA) < 0.08; goodness of fit index (GFI), incremental fit index (IFI), Tucker–Lewis index (TLI), and comparative fit index (CFI) all > 0.90.^[42]^ In addition, CR and AVE were calculated from standardized loadings and error variances to further assess convergent validity, with CR > 0.70 and AVE > 0.50 considered acceptable thresholds.^[43]^

### 2.7. Ethical considerations

This study was approved by the Ethics Committee of Ruikang Hospital Affiliated to Guangxi University of Chinese Medicine (Approval No.: KY2023-046), and pledged adherence to the principles of informed consent, confidentiality, nonmaleficence, strictly following the theoretical principles of the Declaration of Helsinki.

## 3. Results

### 3.1. Delphi expert consultation

#### 3.1.1. Expert characteristics of the sample

Fifteen experts were consulted, most of whom were female (86.7%), aged 40 to 49 years (46.7%), and held a master’s degree (53.3%). The majority had over 20 years of work experience, and 60.0% held vice-senior professional titles. Their expertise spanned clinical nursing, clinical medicine, education, research, and management, with over half (53.3%) serving as graduate supervisors (Table 1).

**Table 1:** Basic information of experts consulted in the survey.

Variables	Category	Number (n = 15)	Percentage (%)
Gender	Male	2	13.33
	Female	13	86.67
Age (yr)	30–39	4	26.66
	40–49	7	46.68
	50–59	4	26.66
Education	Bachelor’s degree	5	33.34
	Master’s degree	8	53.33
	Doctoral degree	2	13.33
Professional title	Vice-senior	9	60.00
	Senior	6	40.00
Years of work experience	10–19	3	20.00
	20–29	7	46.67
	30–39	5	33.33
Main field of expertise	Clinical nursing	4	26.67
	Clinical medicine	2	13.33
	Nursing education	2	13.33
	Nursing research	3	20.00
	Nursing Management	3	20.00
	Psychological nursing	1	6.67
Graduate supervisor	Yes	8	53.33
	No	7	46.67

#### 3.1.2. Expert consultation result

Across 2 rounds of Delphi expert consultation, the participation coefficient for experts was consistently at 100%. Authority coefficients for the first and second rounds were 0.90 and 0.94, respectively. Kendall W coefficients were 0.302 for the first round and 0.325 for the second (*P* < .001), indicating a statistically significant concordance among experts’ ratings. The first round of consultation yielded importance scores for items ranging from 2.67 to 4.67, with variation coefficients spanning 0.104 to 0.337. The second round showed further consensus with item importance scores improving to between 3.73 and 5.00 and variation coefficients reduced to between 0.000 and 0.207.

For the First Round: An expert suggested that the term “COPD” was overly technical, recommending the use of “chronic obstructive pulmonary disease” for clarity. Items with a mean importance value of <3.5 and a CV of 0.25 or higher were eliminated based on the set criteria. As a result, 8 items were removed in this round. Language modifications were made to 4 items, and another 4 items were merged. Additionally, 6 new items were added to the basic knowledge dimension. Ultimately, the revised scale encompassed 5 dimensions with a total of 30 items.

For the Second Round: Seven items underwent language modifications for improved clarity. The data from the second round of expert consultation indicated a consensus among experts, with all items showing a CV <0.25 and a mean importance rating of 3.5 or higher. Hence, further consultation was deemed unnecessary.

### 3.2. Pre-investigation

From this process, 20 questionnaires were retrieved. The findings from the pre-investigation revealed that the average completion time for the scale was 7.32 ± 2.19 minutes. Furthermore, feedback from the patients confirmed that the items were comprehensible and straightforward, eliminating the need for further clarification or alterations. This culminated in the initial draft of the BISPTCM-PR-COPD, which comprises 5 dimensions and 30 items.

### 3.3. Item screening and reliability and validity testing

#### 3.3.1. Demographic characteristics of participants

Considering the clinical context, the first cross-sectional survey in this study distributed 214 questionnaires, successfully retrieving 208 valid responses, achieving a response rate of 97.1%. Details on the general demographics of the study participants are presented in Table 2.

**Table 2:** Demographic characteristics of participants in the first cross-sectional survey.

Characteristic	Category	Number (n = 208)	Percentage (%)
Gender	Male	131	63.0
	Female	77	37.0
Age (yr)	<60	20	9.6
	60–70	85	40.9
	>70	103	49.5
Residence	Urban	128	61.5
	Township	52	25.0
	Rural	28	13.5
Living arrangement	With spouse/partner	72	34.6
	With children	47	22.6
	With extended family	69	33.2
	Alone	20	9.6
Marital status	Married	152	73.1
	Divorced/Widowed	39	18.7
	Single	17	8.2
Education level	Elementary school or below	38	18.3
	Junior high school	96	46.2
	Senior high school/vocational school	45	21.6
	College and above	29	13.9
Employment status	Employed	41	19.7
	Unemployed	70	33.7
	Retired	97	46.6
Monthly household income (CNY)	<2000	35	16.8
	2000–3999	100	48.1
	>4000	73	35.1
Medical expenses payment method	Employee medical insurance	82	39.4
	Resident medical insurance	63	30.3
	New rural cooperative medical scheme	51	24.5
	Self-funded	12	5.8
Duration of COPD (yr)	<5	63	30.3
	5–10	96	46.1
	>10	49	23.6
Number of visits to a doctor for COPD in the last year	1–2	121	58.2
	≥3	87	41.8

COPD = chronic obstructive pulmonary disease.

#### 3.3.2. Item analysis results of the scale

Results of CV method: The lesser the dispersion trend within the scale items, the less distinctive the item is for the subjects assessed. Results revealed that all items in this study’s scale had a SD exceeding 0.75 and a CV surpassing 0.15. Hence, no items were removed from the scale (Table 3).

**Table 3:** Mean, SD, and CV of each item.

Item	Mean (x)	SD	CV	Item	Mean (x)	SD	CV
Q1	3.130	0.977	0.312	Q16	3.471	1.116	0.322
Q2	3.077	1.114	0.362	Q17	3.543	1.107	0.312
Q3	2.995	1.119	0.373	Q18	3.654	1.088	0.298
Q4	2.976	1.014	0.341	Q19	3.611	1.034	0.286
Q5	2.918	1.080	0.370	Q20	3.317	1.066	0.321
Q6	2.984	0.899	0.323	Q21	3.635	0.858	0.236
Q7	2.947	1.027	0.349	Q22	3.591	1.008	0.281
Q8	2.986	0.955	0.320	Q23	3.601	1.016	0.282
Q9	3.683	0.920	0.250	Q24	3.663	0.964	0.263
Q10	3.563	0.893	0.251	Q25	3.538	1.035	0.292
Q11	3.538	0.905	0.256	Q26	3.596	0.885	0.246
Q12	3.639	0.857	0.235	Q27	3.659	1.173	0.321
Q13	3.615	0.904	0.250	Q28	3.558	1.190	0.335
Q14	3.601	1.026	0.285	Q29	3.697	1.129	0.305
Q15	3.606	0.942	0.261	Q30	3.755	1.073	0.286

CV = coefficient of variation (SD/Mean); SD = standard deviation.

Results of Critical Ratio method: For each completed scale, total scores were calculated and ranked from highest to lowest. The top 27% of scores were categorized as the high score group, and the bottom 27% as the low score group. An independent samples *t*-test was used to compare the score differences between these 2 groups. The results demonstrated statistically significant differences in scores between the groups (*P* < .001), with no items meeting the criteria for removal (Table 4).

**Table 4:** Results of critical ratio analysis.

Item	*t*-Value	*P*-value	Item	*t*-Value	*P*-value
Q1	−7.044	<.001	Q16	−12.791	<.001
Q2	−7.112	<.001	Q17	−12.200	<.001
Q3	−5.721	<.001	Q18	−14.199	<.001
Q4	−8.617	<.001	Q19	−13.413	<.001
Q5	−6.285	<.001	Q20	−12.656	<.001
Q6	−4.423	<.001	Q21	−11.784	<.001
Q7	−3.683	<.001	Q22	−12.290	<.001
Q8	−3.327	<.001	Q23	−11.916	<.001
Q9	−10.854	<.001	Q24	−10.981	<.001
Q10	−9.698	<.001	Q25	−9.803	<.001
Q11	−9.253	<.001	Q26	−11.693	<.001
Q12	−9.617	<.001	Q27	−16.442	<.001
Q13	−11.242	<.001	Q28	−15.981	<.001
Q14	−13.392	<.001	Q29	−17.756	<.001
Q15	−11.370	<.001	Q30	−18.156	<.001

Results of item-total correlation coefficient method: The correlation coefficients (*r*) between item scores and the total score of the scale ranged from 0.181 to 0.803. Specifically, the *r* values for items Q7 and Q8 were 0.253 and 0.181, respectively. These *r* values, being <0.3, indicate a weak correlation with the total scale score, suggesting consideration for removal (Table 5).

**Table 5:** Results of item-total correlation coefficient method.

Item	Item-total correlation (*r*)	*P*-value	Item	Item-total correlation (*r*)	*P*-value
Q1	0.415	<.001	Q16	0.742	<.001
Q2	0.359	<.001	Q17	0.744	<.001
Q3	0.421	<.001	Q18	0.788	<.001
Q4	0.531	<.001	Q19	0.764	<.001
Q5	0.354	<.001	Q20	0.741	<.001
Q6	0.313	<.001	Q21	0.624	<.001
Q7	*0.253*	<.001	Q22	0.711	<.001
Q8	*0.181*	<.001	Q23	0.723	<.001
Q9	0.720	<.001	Q24	0.651	<.001
Q10	0.694	<.001	Q25	0.724	<.001
Q11	0.645	<.001	Q26	0.673	<.<.001
Q12	0.712	<.001	Q27	0.784	<.001
Q13	0.761	<.001	Q28	0.789	<.001
Q14	0.784	<.001	Q29	0.773	<.001
Q15	0.724	<.001	Q30	0.803	<.001

The italicized values in the table indicates that the item does not meet the screening criteria.

Results of the internal consistency coefficient method: For the overall scale, the Cronbach alpha coefficient is 0.947. After the removal of items Q2, Q5, Q6, Q7, and Q8, the Cronbach alpha coefficient for the overall scale increased, indicating these items reduced the internal consistency of the scale and should be considered for removal (Table 6).

**Table 6:** Cronbach α if item deleted.

Item	Cronbach α if item deleted	Change	Item	Cronbach α if item deleted	Change
Q1	0.947	↓	Q16	0.944	↓
Q2	*0.948*	↑	Q17	0.944	↓
Q3	0.947	↓	Q18	0.943	↓
Q4	0.946	↓	Q19	0.944	↓
Q5	*0.948*	↑	Q20	0.944	↓
Q6	*0.948*	↑	Q21	0.945	↓
Q7	*0.949*	↑	Q22	0.944	↓
Q8	*0.949*	↑	Q23	0.944	↓
Q9	0.944	↓	Q24	0.945	↓
Q10	0.944	↓	Q25	0.944	↓
Q11	0.945	↓	Q26	0.945	↓
Q12	0.944	↓	Q27	0.943	↓
Q13	0.944	↓	Q28	0.943	↓
Q14	0.943	↓	Q29	0.943	↓
Q15	0.944	↓	Q30	0.943	↓

The italicized values indicate items whose deletion increased the Cronbach’s alpha coefficient (i.e., these items reduced the internal consistency of the scale). They do not represent statistical significance. ↑ indicates that Cronbach α increased if the item was deleted; ↓ indicates that Cronbach α decreased.

Summary and analysis of item screening results: The selection of items was conducted using 4 methods, with items not meeting the established criteria on 2 or more occasions being removed. Specifically, items Q7 and Q8, which failed to meet both the item-total correlation and Internal Consistency Coefficient methods, were excluded. The EFA then proceeded with the remaining 28 items.

#### 3.3.3. EFA results of the scale

The KMO value of Sample N1 (n = 208) was 0.925, and Bartlett test of sphericity was significant (χ^2^ = 5117.826, df = 378, *P* < .001), indicating that the data were suitable for factor analysis. EFA was performed using principal axis factoring with oblique rotation (direct oblimin), as the theoretical dimensions of the TPB were expected to be correlated. Five factors with eigenvalues >1 were extracted, accounting for a cumulative variance of 64.545%. The factor correlation matrix indicated moderate inter-factor correlations (r = –0.67 to 0.55), supporting the use of oblique rotation. Inspection of the Pattern Matrix revealed that the item “I am aware of the characteristics of Yijin Jing, which involves stretching movements and tendon extension” had a factor loading below 0.40, and the item “I believe the methods of TCM exercises for pulmonary rehabilitation are simple and easy to master” did not align with the expected factors or the content of any existing factor. After discussion by the research team, these 2 items were removed prior to the second EFA.

**Figure 2.**
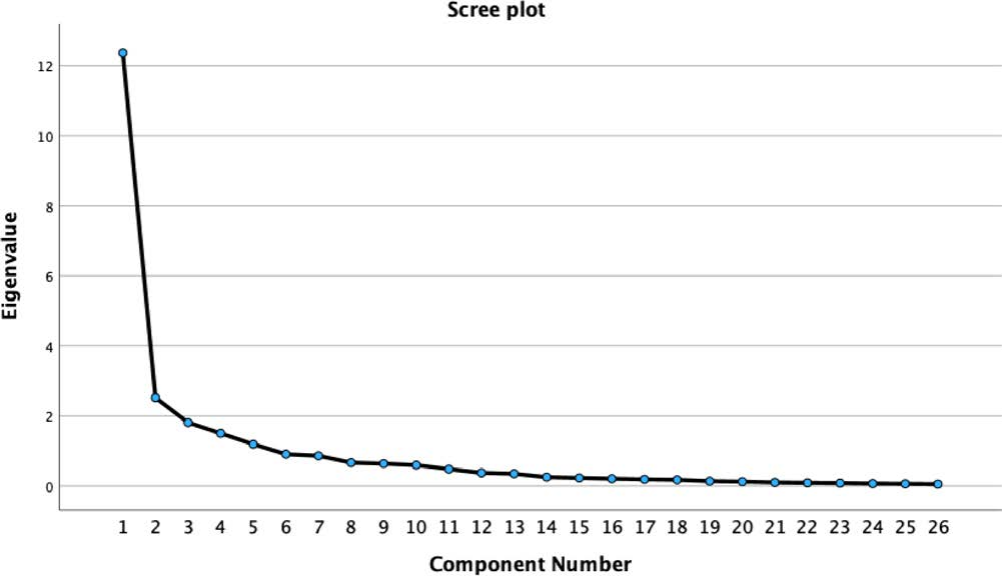
Scree plot.

After eliminating 2 items, a second EFA was conducted on the remaining 26 items. The KMO measure was 0.920, and Bartlett test of sphericity was significant (χ^2^ = 5432.21, df = 325, *P* < .001), confirming the adequacy of the data for factor analysis. Using principal axis factoring with oblique rotation (direct oblimin), 5 factors with eigenvalues >1 were extracted, which together explained 68.908% of the total variance. The scree plot further supported the retention of 5 factors. Examination of the Pattern Matrix showed that all items had loadings above 0.40 on their respective factors without cross-loadings. The factor structure was consistent with theoretical expectations, and the 5 factors were labeled as basic knowledge, attitudes, subjective norms, perceived behavioral control, and behavioral intention. The final version of the scale therefore comprised 26 items across these 5 dimensions (see Fig. 2 and Table 7).

**Table 7:** EFA results for TCM exercises for pulmonary rehabilitation.

Items	Common factors				
	Subjective norms	Attitudes	Perceived behavioral control	Basic knowledge	Behavioral intention
Recommendations from authoritative respiratory experts would prompt me to engage in traditional Chinese medicine exercises for pulmonary rehabilitation.	0.914				
Support from my friends and family would encourage me to engage in traditional Chinese medicine exercises for pulmonary rehabilitation.	0.887				
Promotions on social media networks would motivate me to engage in traditional Chinese medicine exercises for pulmonary rehabilitation.	0.863				
Seeing other patients undergoing traditional Chinese medicine exercises for pulmonary rehabilitation would encourage me to participate too.	0.808				
Guidance and suggestions from healthcare professionals would encourage me to engage in traditional Chinese medicine exercises for pulmonary rehabilitation.	0.688				
I believe traditional Chinese medicine exercises for pulmonary rehabilitation can improve my quality of life.		0.919			
I believe traditional Chinese medicine exercises for pulmonary rehabilitation can alleviate symptoms such as coughing, expectoration, and breathlessness.		0.896			
I consider traditional Chinese medicine exercises for pulmonary rehabilitation a good nonpharmacological treatment method.		0.874			
I believe traditional Chinese medicine exercises for pulmonary rehabilitation can reduce the frequency of acute chronic obstructive pulmonary disease exacerbations.		0.792			
I believe traditional Chinese medicine exercises for pulmonary rehabilitation can ease my feelings of anxiety and depression.		0.772			
I am very interested in traditional Chinese medicine exercises for pulmonary rehabilitation.		0.723			
I can decide whether to engage in traditional Chinese medicine exercises for pulmonary rehabilitation on my own.			0.902		
I have sufficient time to engage in traditional Chinese medicine exercises for pulmonary rehabilitation.			0.891		
I would overcome any difficulties to participate in traditional Chinese medicine exercises for pulmonary rehabilitation.			0.625		
I have the financial ability to engage in traditional Chinese medicine exercises for pulmonary rehabilitation.			0.619		
I have enough perseverance to continue participating in traditional Chinese medicine exercises for pulmonary rehabilitation.			0.597		
My physical condition allows me to engage in traditional Chinese medicine exercises for pulmonary rehabilitation.			0.431		
I know that exhalation and vocalization are unique practices in the Liuzijue.				0.604	
I am aware that chronic obstructive pulmonary disease patients should gradually increase the intensity and duration of traditional Chinese medicine exercises for pulmonary rehabilitation.				0.571	
I know the Baduanjin consists of 8 different movements.				0.554	
I understand that chronic obstructive pulmonary disease patients cannot engage in traditional Chinese medicine exercises for pulmonary rehabilitation at any time.				0.530	
I am aware that Tai Chi not only demands mental focus but also requires specific breathing techniques.				0.492	
I plan to engage in traditional Chinese medicine exercises for pulmonary rehabilitation in the future					0.942
I am willing to introduce the benefits of traditional Chinese medicine exercises for pulmonary rehabilitation to others.					0.876
I plan to actively seek out information about traditional Chinese medicine exercises for pulmonary rehabilitation					0.874
I currently have plans to engage in traditional Chinese medicine exercises for pulmonary rehabilitation					0.843
Eigenvalue	12.359	2.520	1.809	1.502	1.190
Variance contribution (%)	47.535	9.691	6.958	5.776	4.578
Cumulative variance contribution (%)	47.535	57.227	64.185	69.961	74.539

EFA = exploratory factor analysis, TCM = traditional Chinese medicine.

#### 3.3.4. Reliability analysis results of the scale

The scale demonstrated high reliability. The overall Cronbach α was 0.951, with dimension-specific coefficients ranging from 0.711 to 0.970, all exceeding the acceptable threshold of 0.60. Split-half reliability was 0.883 for the total scale, with values across dimensions ranging from 0.743 to 0.976. Test–retest reliability, assessed by ICC, was also satisfactory, with an overall ICC of 0.927 (95% CI: 0.86–0.97) and dimension-specific ICCs ranging from 0.781 to 0.915. Collectively, these findings support the scale’s good internal consistency and temporal stability (Table 8).

**Table 8:** Reliability coefficients of the total scale and each dimension.

Dimension	Cronbach α	Split-half reliability	Test–retest (ICC, 95% CI)
Basic knowledge	0.711	0.743	0.781 (95% CI: 0.61–0.89)
Attitudes	0.947	0.952	0.842 (95% CI: 0.70–0.92)
Subjective norms	0.963	0.965	0.868 (95% CI: 0.74–0.94)
Perceived behavioral control	0.895	0.937	0.893 (95% CI: 0.80–0.96)
Behavioral intention	0.970	0.976	0.915 (95% CI: 0.84–0.96)
Total	0.951	0.883	0.927 (95% CI: 0.86–0.97)

ICC = intraclass correlation coefficient.

#### 3.3.5. Content validity analysis results of the scale

For the second round of expert consultation, 15 experts were invited to assess the content validity of the scale. Based on the relevance scores provided by the experts, the I-CVI was calculated for each item, with scores ranging from 0.8 to 1.00, all meeting or exceeding the threshold of 0.78. The S-CVI was calculated to be 0.933, surpassing the acceptability criterion of 0.9. This indicates that the scale’s content is acceptable and accurately reflects the research topic.

#### 3.3.6. CFA results of the scale

Demographic characteristics of participants: Based on clinical realities, the second cross-sectional survey in this study distributed a total of 230 questionnaires, with 222 being effectively collected, yielding a valid response rate of 96.5%. Details on the general demographics of the study participants are presented in Table 9.

**Table 9:** Demographic characteristics of participants in the second cross-sectional survey.

Characteristic	Category	Number (n = 222)	Percentage (%)
Gender	Male	143	64.4
	Female	79	35.6
Age (yr)	<60	37	16.7
	60–70	89	40.1
	>70	96	43.2
Residence	Urban	146	65.8
	Township	48	21.6
	Rural	28	12.6
Living arrangement	With spouse/partner	93	41.9
	With children	45	20.3
	With extended family	66	29.7
	Alone	18	8.1
Marital status	Married	170	76.6
	Divorced/widowed	38	17.1
	Single	14	6.3
Education level	Elementary school or below	33	14.9
	Junior high school	92	41.4
	Senior high school/vocational school	61	27.5
	College and above	36	16.2
Employment status	Employed	57	25.7
	Unemployed	71	32.0
	Retired	94	42.3
Monthly household income (CNY)	<2000	35	15.8
	2000–3999	101	45.5
	>4000	86	38.7
Medical expenses payment method	Employee medical insurance	98	44.1
	Resident medical insurance	60	27.1
	New rural cooperative medical scheme	48	21.6
	Self-funded	16	7.2
Duration of COPD (yr)	<5	81	36.5
	5–10	98	44.1
	>10	43	19.4
Number of visits to a doctor for COPD in the last year	1–2	132	59.5
	≥3	90	40.5

COPD = chronic obstructive pulmonary disease.

**Figure 3.**
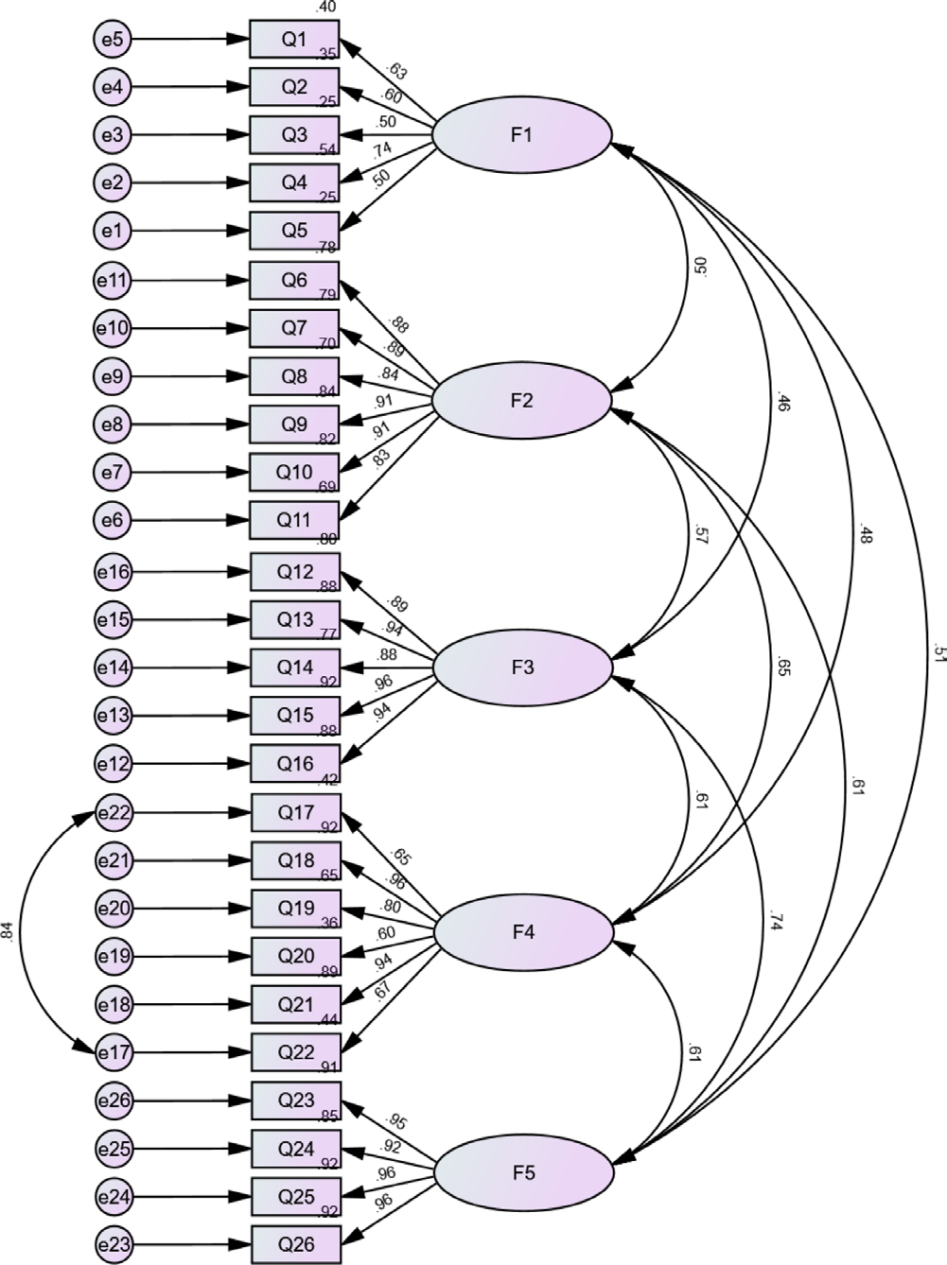
Path diagram of the confirmatory factor analysis. Q1 to Q23 represents the items of BISPTCM-PR-COPD, F1 to F5 represents the 5 factors of BISPTCM-PR-COPD. BISPTCM-PR-COPD = behavioral intention scale for pulmonary rehabilitation with traditional Chinese medicine in COPD patients, COPD = chronic obstructive pulmonary disease.

The initial results of the CFA of Sample N2 (n = 222) in this study indicated a high correlation between the residuals of items Q17 and Q22. A second CFA was conducted by adding a covariance link between the error residuals of these 2 items. The model fit indices were as follows: χ^2^/df = 2.259 (<3.000), RMSEA = 0.075 (<0.08), IFI = 0.941 (>0.90), TLI = 0.933 (>0.90), CFI = 0.941 (>0.90), and GFI = 0.822. These indices suggest that the scale’s structural validity has reached an acceptable level (Fig. 3).

#### 3.3.7. Convergent validity

CR and AVE were calculated to assess convergent validity. CR values ranged from 0.735 to 0.973, all above the 0.70 threshold. AVE values ranged from 0.362 to 0.901; except for basic knowledge (AVE = 0.362), all factors exceeded the 0.50 cutoff, indicating satisfactory convergent validity. The results are presented in Table 10.

**Table 10:** Composite reliability and average variance extracted of the 5 factors.

Factor	CR	AVE
Basic knowledge	0.735	0.362
Attitudes	0.952	0.769
Subjective norms	0.966	0.851
Perceived behavioral control	0.902	0.613
Behavioral intention	0.973	0.901

AVE = average variance extracted, CR = composite reliability.

## 4. Discussion

### 4.1. Scientific and normative development process

The BISPTCM-PR-COPD was developed following recognized standards for scale construction, ensuring scientific rigor and methodological credibility. Guided by the extended TPB, its development combined a comprehensive literature review with qualitative interviews, enabling the scale to reflect both theoretical foundations and patients’ lived experiences. Expert consultations with professionals in TCM, COPD, and pulmonary rehabilitation further enhanced the cultural appropriateness and clinical relevance of the items.

This iterative process – spanning item generation, expert review, pilot testing, and psychometric evaluation – ensured that the instrument was repeatedly refined rather than mechanically tested. Items were adjusted for clarity, cultural fit, and clinical applicability, which contributed to the robustness of the final version. The resulting 26-item, 5-dimension scale demonstrates strong methodological grounding and also offers a reference model for developing similar instruments in other culturally embedded health care contexts.

### 4.2. Reliability and validity of the BISPTCM-PR-COPD

The BISPTCM-PR-COPD demonstrated good reliability across multiple indicators. Internal consistency was high, with the overall Cronbach alpha reaching 0.951, and most dimensions exceeding 0.80. One domain, however, showed a relatively lower alpha of 0.711, which still meets the minimum acceptable standard but suggests greater heterogeneity of items. Clinically, this indicates that while the domain is consistent, patient responses may vary more widely, warranting further refinement in future iterations. Split-half reliability (0.883 overall) and test–retest reliability (ICC = 0.927) further confirmed the stability of the instrument, underscoring its dependability in repeated applications.

Validity testing also yielded positive results. Expert evaluations supported strong content validity (S-CVI = 0.933), while EFA confirmed the expected 5-factor structure with a cumulative variance contribution of 68.9%. All factor loadings exceeded 0.40, and the rotated structure closely aligned with theoretical expectations. In CFA, most model fit indices reached recommended thresholds, while the GFI (0.822) was below the conventional cutoff of 0.90. To improve model fit, a covariance was added between the residuals of 2 perceived behavioral control items (Q17 and Q22). This adjustment was theoretically justified, as both items reflect barriers to participation: one emphasizes financial constraints and the other emphasizes perseverance in overcoming difficulties. Their correlation highlights the interplay of socioeconomic and psychological resources in shaping patient behavior, rather than representing a purely data-driven modification.

When examining convergent validity, most factors achieved satisfactory CR (0.902 to 0.973) and AVE (0.55–0.90). However, the basic knowledge domain showed a lower AVE (0.362) despite an acceptable CR (0.735). This suggests that while the items were internally consistent, they may not have captured sufficient shared variance. Possible reasons include the heterogeneous nature of patients’ knowledge levels and the relatively broad scope of knowledge items. For clinical use, low scores in this domain highlight patients who lack fundamental understanding of TCM pulmonary rehabilitation and may require tailored educational interventions before meaningful attitudes or intention changes can occur. Addressing this gap could enhance both patient engagement and the predictive validity of the scale.

### 4.3. Implications for clinical practice

The BISPTCM-PR-COPD is not only a psychometric tool for research but also holds important clinical value. By quantifying patients’ intentions and identifying specific psychosocial determinants, the scale can serve as a screening instrument in clinical and community settings to inform tailored interventions. Patients with low scores may face greater barriers to participation in pulmonary rehabilitation and could therefore benefit from targeted education, motivational support, or resource-based interventions provided at an early stage.^[44]^

Equally important, examining scores across individual domains provides actionable insights. For instance, low scores in the basic knowledge domain suggest insufficient understanding of TCM exercise principles, indicating the need for structured health education before patients can form positive attitudes or confidence.^[45]^ Low scores in Attitudes may call for motivational interviewing or tailored counseling to address misconceptions,^[46]^ while deficits in subjective norms highlight the need to mobilize family support and peer encouragement.^[47]^ Similarly, low scores in perceived behavioral control reflect practical or economic barriers; addressing these may require financial counseling, resource referral, or simplifying the exercise protocols.^[48]^

By pinpointing the domains in which patients struggle, the scale enables healthcare providers to design personalized care strategies – whether through educational materials, family involvement, or multidisciplinary support – rather than applying one-size-fits-all approaches.^[49]^ In this way, the BISPTCM-PR-COPD bridges the gap between research and practice, offering a structured pathway to optimize patient engagement and ultimately improve the effectiveness of TCM pulmonary rehabilitation in COPD management.

## 5. Limitations

This study has several limitations. First, the scale relied on patient self-report, which may be subject to recall and social desirability bias. Second, participants were recruited from a single province using convenience sampling, limiting representativeness; larger and multi-center samples are needed to improve generalizability. Third, although CFA showed acceptable fit overall, the GFI fell below the optimal cutoff and some error terms were correlated, indicating that further refinement is warranted. Fourth, the basic knowledge domain yielded a relatively low AVE, suggesting that item revision may be needed to strengthen convergent validity. Fifth, the study only assessed cross-sectional reliability and validity; responsiveness to change and predictive validity were not examined and should be tested in future longitudinal or intervention studies. Finally, as the scale was developed in the Chinese healthcare and cultural context, its applicability outside China remains uncertain and will require cross-cultural adaptation and validation.

## 6. Conclusion

To our knowledge, this study is the first to develop and validate a scale specifically assessing the behavioral intentions of COPD patients to participate in TCM exercises for pulmonary rehabilitation. Grounded in the extended TPB, the BISPTCM-PR-COPD demonstrated good reliability and validity, providing a psychometrically sound instrument for this unique cultural and clinical context. Beyond its methodological contribution, the scale offers practical value: it can be used to screen patients with low readiness to engage, identify specific psychosocial barriers across different domains, and inform tailored interventions. By doing so, it bridges the gap between research and clinical practice, supporting more effective implementation of TCM pulmonary rehabilitation. Future studies should further test its predictive validity, responsiveness, and applicability across broader and cross-cultural populations.

## Acknowledgments

We thank Home for Researchers editorial team (www.home-for-researchers.com) for language editing service.

## Author contributions

**Conceptualization:** Yuyin Chen, Meijiang Chen, Xiuhong Long.

**Data curation:** Yuyin Chen, Huiqiong Tu, Yuanyuan Zhang, Shujin Cheng.

**Formal analysis:** Yuyin Chen, Meijiang Chen, Jie Jin, Yuhua Qiu.

**Writing – original draft:** Yuyin Chen, Meijiang Chen, Huiqiong Tu, Wanlin Peng.

**Writing – review & editing:** Yuyin Chen, Meijiang Chen, Xiuhong Long, Yuanyuan Zhang, Jie Jin.

## Supplementary Material


