# Supplementary material for: Development and validation of the behavioral intention scale for participation in traditional Chinese medicine exercises for pulmonary rehabilitation among COPD patients
Source: Medicine (Baltimore). 2025 Oct 31;104(44):e45336. doi: 10.1097/MD.0000000000045336 (PMC12582716; doi:10.1097/MD.0000000000045336)
Supplement: Supplementary file 1 [file medi-104-e45336-s001.pdf]

Appendix

Behavioral Intention Scale for Participation in Traditional Chinese Medicine Exercises for Pulmonary Rehabilitation among COPD Patients

Guidance note: Below are important questions related to traditional Chinese medicine exercises for pulmonary rehabilitation. Five numbers are provided for each question; please circle the number you think most closely matches your feeling.					
Basic Knowledge	Completely Unaware	Slightly Aware	Unsure	Mostly Aware	Fully Aware
1. I understand that COPD patients should not practice TCM pulmonary rehabilitation exercises at inappropriate times.	1	2	3	4	5
2. I am aware that chronic obstructive pulmonary disease patients should gradually increase the intensity and duration of traditional Chinese medicine exercises for pulmonary rehabilitation.	1	2	3	4	5
3. I am aware that Tai Chi not only demands mental focus but also requires specific breathing techniques.	1	2	3	4	5
4. I know the Baduanjin consists of eight distinct movements.	1	2	3	4	5
5. I know that exhalation and vocalization are unique practices in the Liuzijue.	1	2	3	4	5
Attitudes	Strongly Disagree	Disagree	Neither agree nor disagree	Agree	Strongly Agree
1. I consider traditional Chinese medicine exercises for pulmonary rehabilitation a good non-pharmacological treatment method.	1	2	3	4	5
2. I believe traditional Chinese medicine exercises for pulmonary rehabilitation can alleviate symptoms such as coughing, expectoration, and breathlessness.	1	2	3	4	5
3. I believe traditional Chinese medicine exercises for pulmonary rehabilitation can reduce the frequency of acute chronic obstructive pulmonary disease exacerbations.	1	2	3	4	5
4. I believe traditional Chinese medicine exercises for	1	2	3	4	5

pulmonary rehabilitation can improve my quality of life.					
5. I believe traditional Chinese medicine exercises for pulmonary rehabilitation can ease my feelings of anxiety and depression.	1	2	3	4	5
6. I am very interested in traditional Chinese medicine exercises for pulmonary rehabilitation.	1	2	3	4	5
Subjective Norms	Strongly Disagree	Disagree	Neither agree nor disagree	Agree	Strongly Agree
1. Seeing other patients undergoing traditional Chinese medicine exercises for pulmonary rehabilitation would encourage me to participate too.	1	2	3	4	5
2. Support from my friends and family would encourage me to engage in traditional Chinese medicine exercises for pulmonary rehabilitation.	1	2	3	4	5
3. Guidance and suggestions from healthcare professionals would encourage me to engage in traditional Chinese medicine exercises for pulmonary rehabilitation.	1	2	3	4	5
4. Recommendations from authoritative respiratory experts would prompt me to engage in traditional Chinese medicine exercises for pulmonary rehabilitation.	1	2	3	4	5
5. Health information on media (such as television, newspapers, or social media) would motivate me to engage in traditional Chinese medicine exercises for pulmonary rehabilitation.	1	2	3	4	5
Perceived Behavioral Control	Strongly Disagree	Disagree	Neither agree nor disagree	Agree	Strongly Agree
1. I have the financial ability to engage in traditional Chinese medicine exercises for pulmonary rehabilitation.	1	2	3	4	5
2. I have sufficient time to engage in traditional Chinese medicine exercises for pulmonary rehabilitation.	1	2	3	4	5
3. I have enough perseverance to continue participating in traditional Chinese medicine exercises for pulmonary	1	2	3	4	5

rehabilitation.					
4. My physical condition allows me to engage in traditional Chinese medicine exercises for pulmonary rehabilitation.	1	2	3	4	5
5. I am confident that I can decide independently whether to engage in traditional Chinese medicine exercises for pulmonary rehabilitation.	1	2	3	4	5
6. I would overcome any difficulties to participate in traditional Chinese medicine exercises for pulmonary rehabilitation.	1	2	3	4	5
Behavioral Intention	Strongly Disagree	Disagree	Neither agree nor disagree	Agree	Strongly Agree
1. I plan to actively seek out information about traditional Chinese medicine exercises for pulmonary rehabilitation	1	2	3	4	5
2. I currently have plans to engage in traditional Chinese medicine exercises for pulmonary rehabilitation	1	2	3	4	5
3. I plan to engage in traditional Chinese medicine exercises for pulmonary rehabilitation in the future	1	2	3	4	5
4. I am willing to introduce the benefits of traditional Chinese medicine exercises for pulmonary rehabilitation to others.	1	2	3	4	5
